# Improving Suspected Pulmonary Infection Diagnosis by Bronchoalveolar Lavage Fluid Metagenomic Next-Generation Sequencing: a Multicenter Retrospective Study

**DOI:** 10.1128/spectrum.02473-21

**Published:** 2022-08-09

**Authors:** Xiao Jin, Juan Li, Mingyue Shao, Xuedong Lv, Ningfei Ji, Yehan Zhu, Mao Huang, Feichao Yu, Changwen Zhang, Lixu Xie, Jianling Huang, Sixi Chen, Changjun Zhu, Minjie Lv, Ganzhu Feng

**Affiliations:** a Department of Respiratory Medicine, The Second Clinical Medical School of Nanjing Medical University, Nanjing, Jiangsu, China; b Department of Pulmonary and Critical Care Medicine, The Second Affiliated Hospital of Nanjing Medical University, Nanjing, Jiangsu, China; c Department of Pulmonary and Critical Care Medicine, The Second Affiliated Hospital of Nantong University, Nantong, China; d Department of Pulmonary and Critical Care Medicine, The First Affiliated Hospital of Nanjing Medical University, Nanjing, Jiangsu, China; e Department of Pulmonary and Critical Care Medicine, The First Affiliated Hospital of Soochow University, Suzhou, China; Emory University School of Medicine

**Keywords:** mNGS, pulmonary infection, pathogen, BALF

## Abstract

Metagenomic next-generation sequencing (mNGS) has been gradually applied to clinical practice due to its unbiased characteristics of pathogen detection. However, its diagnostic performance and clinical value in suspected pulmonary infection need to be evaluated. We systematically reviewed the clinical data of 246 patients with suspected pulmonary infection from 4 medical institutions between January 2019 and September 2021. The diagnostic performances of mNGS and conventional testing (CT) were systematically analyzed based on bronchoalveolar lavage fluid (BALF). The impacts of mNGS and CT on diagnosis modification and treatment adjustment were also assessed. The positive rates of mNGS and CT were 47.97% and 23.17%, respectively. The sensitivity of mNGS was significantly higher than that of CT (53.49% versus 23.26%, *P* < 0.01), especially for infections of Mycobacterium tuberculosis (67.86% versus 17.86%, *P* < 0.01), atypical pathogens (100.00% versus 7.14%, *P* < 0.01), viruses (92.31% versus 7.69%, *P* < 0.01), and fungi (78.57% versus 39.29%, *P* < 0.01). The specificity of mNGS was superior to that of CT, with no statistical difference (90.32% versus 77.42%, *P* = 0.167). The positive predictive value (PPV) and negative predictive value (NPV) of mNGS were 97.46% and 21.88%, respectively. Diagnosis modification and treatment adjustment were conducted in 32 (32/246, 13.01%) and 23 (23/246, 9.35%) cases, respectively, according to mNGS results only. mNGS significantly improved the diagnosis of suspected pulmonary infection, especially infections of M. tuberculosis, atypical pathogens, viruses, and fungi, and it demonstrated the pathogen distribution of pulmonary infections. It is expected to be a promising microbiological detection and diagnostic method in clinical practice.

**IMPORTANCE** Pulmonary infection is a heterogeneous and complex infectious disease with high morbidity and mortality worldwide. In clinical practice, a considerable proportion of the etiology of pulmonary infection is unclear, microbiological diagnosis being challenging. Metagenomic next-generation sequencing detects all nucleic acids in a sample in an unbiased manner, revealing the microbial community environment and organisms and improving the microbiological detection and diagnosis of infectious diseases in clinical settings. This study is the first multicenter, large-scale retrospective study based entirely on BALF for pathogen detection by mNGS, and it demonstrated the superior performance of mNGS for microbiological detection and diagnosis of suspected pulmonary infection, especially in infections of Mycobacterium tuberculosis, atypical pathogens, viruses, and fungi. It also demonstrated the pathogen distribution of pulmonary infections in the real world, guiding targeted treatment and improving clinical management and prognoses.

## INTRODUCTION

Pulmonary infection is heterogeneous, complex, and the most common infectious disease, with high morbidity and mortality worldwide (1), and 19% to 62% of its etiology is unclear in clinical practice (2, 3), presenting challenges for microbiological diagnosis.

Currently, conventional testing (CT) for pathogen detection widely applied in clinical settings mainly includes microbial culture, antigen/antibody assay, and PCR-based nucleic acid detection, but the diagnostic efficiencies of these methods need to be improved (4). Cultures of common bacteria or fungi generally take 3 to 7 days (5, 6). Mycobacterium tuberculosis grows slowly and requires special nutritional components, and cultures usually take about 45 days (7). Besides this, the positive rate of pathogen detection for CT, especially microbial culture, may be severely affected by previous antimicrobial therapy (8). The application range of antigen/antibody assays is limited, and their results are often affected by thresholds (9). Despite its high sensitivity and specificity, the implementation of PCR requires the prediction of pathogens to design corresponding primers (10). The deficiencies of CT usually lead to delayed diagnosis, misdiagnosis, and even inappropriate use of antibiotics (11). Obviously, it is necessary to apply new, superior methods for pathogen detection and diagnosis confirmation, guiding targeted treatment and improving prognoses.

Metagenomic next-generation sequencing (mNGS), also known as unbiased NGS or clinical mNGS, is a method for parallel sequencing of all nucleic acids in a sample (12). mNGS has a broad detection spectrum, including bacteria, viruses, fungi, atypical pathogens, parasites, and even new microorganisms. In addition, the diagnostic efficiency of mNGS is hardly affected by antibiotics (7). mNGS has been applied in clinical settings such as the diagnosis of infectious diseases and the acquisition of novel microbial genome sequences (13–15), emerging as a promising single, universal pathogen detection method for infectious disease diagnosis and tracking. Although a review of the diagnostic value of mNGS in lower respiratory tract infection has shown that mNGS has some advantages over CT in detecting pathogens, several hurdles need to be addressed, such as differentiation of colonization from infection, extraneous sources of nucleic acid, method standardization, and data storage, protection, analysis, and interpretation (12, 16). The diagnostic efficiency and clinical practice value of mNGS in suspected pulmonary infection still needs to be explored.

This is a multicenter, large-scale, comprehensive retrospective clinical study. We reviewed the clinical data of 400 patients with suspected pulmonary infection from 4 medical institutions in China between January 2019 and September 2021 and ultimately enrolled 246 cases, exploring the diagnostic performances of mNGS and CT and the pathogen distribution of pulmonary infection, and investigating the clinical application value of mNGS in suspected pulmonary infection.

## RESULTS

### Sample and patient characteristics.

**FIG 1:**
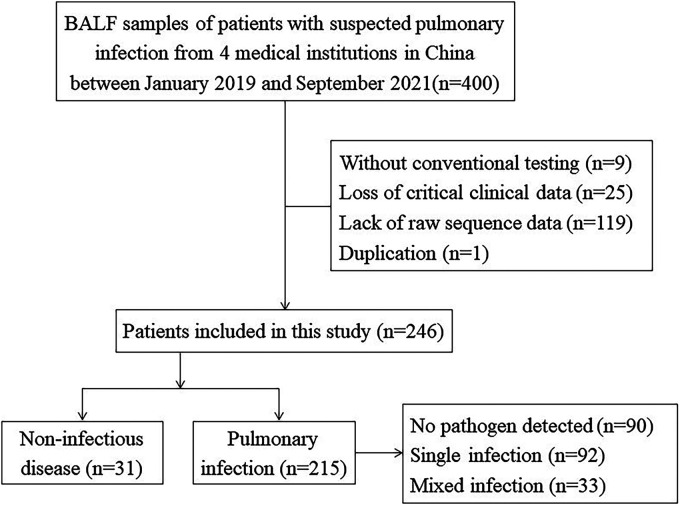
Flow diagram of the study. A total of 400 bronchoalveolar lavage fluid (BALF) samples of patients with suspected pulmonary infection from 4 medical institutions in China between January 2019 and September 2021 were reviewed and eventually 246 cases were included in the study. All cases were examined by metagenomic next-generation sequencing (mNGS) and conventional testing (CT) and were eventually diagnosed as non-infectious diseases or pulmonary infection.

Between January 2019 and September 2021, 400 patients with suspected pulmonary infection from 4 medical institutions in China were initially enrolled in this study. All patients underwent bronchoscopy and bronchoalveolar lavage fluid (BALF) was collected for mNGS and CT. A total of 154 patients were excluded due to lack of paired CT (*n* = 9), loss of key clinical data (*n* = 25), lack of raw sequence data (*n* = 119), and duplication (*n* = 1). Eventually, a total of 246 patients were included (Fig. 1), 159 men and 87 women, with a median age of 60 years. Among these, a total of 156 patients had at least one comorbidity, involving respiratory system diseases (69/246, 28.05%), circulatory system diseases (70/246, 28.46%), metabolic diseases (35/246, 14.23%), kidney diseases (3/246, 1.22%), neurological diseases (5/246, 2.03%), autoimmune diseases (14/246, 5.69%), tumors (41/246, 16.67%), and mental system diseases (7/246, 2.85%). The majority of patients (235/246, 95.53%) were exposed to antibiotics (received within 72 h) prior to sampling. In total, the median hospital stay was 14 days (Table 1).

**TABLE 1:** Baseline characteristics of 246 patients included^*a*^

Characteristic	*N*	%
Gender		
Male	159	64.63
Female	87	35.37
Age (yrs)		
≥60	125	50.81
<60	121	49.19
Comorbidities		
Respiratory diseases	69	28.05
Circulatory diseases	70	28.46
Metabolic diseases	35	14.23
Renal diseases	3	1.22
Neurological diseases	5	2.03
Autoimmune diseases	14	5.69
Tumors	41	16.67
Mental diseases	7	2.85
Antibiotic exposure before mNGS		
Yes	235	95.53
No	11	4.47
Hospital stays (days)		
≥14	128	52.03
<14	118	47.97

a mNGS, metagenomic next-generation sequencing.

### Concordance of mNGS and CT.

**FIG 2:**
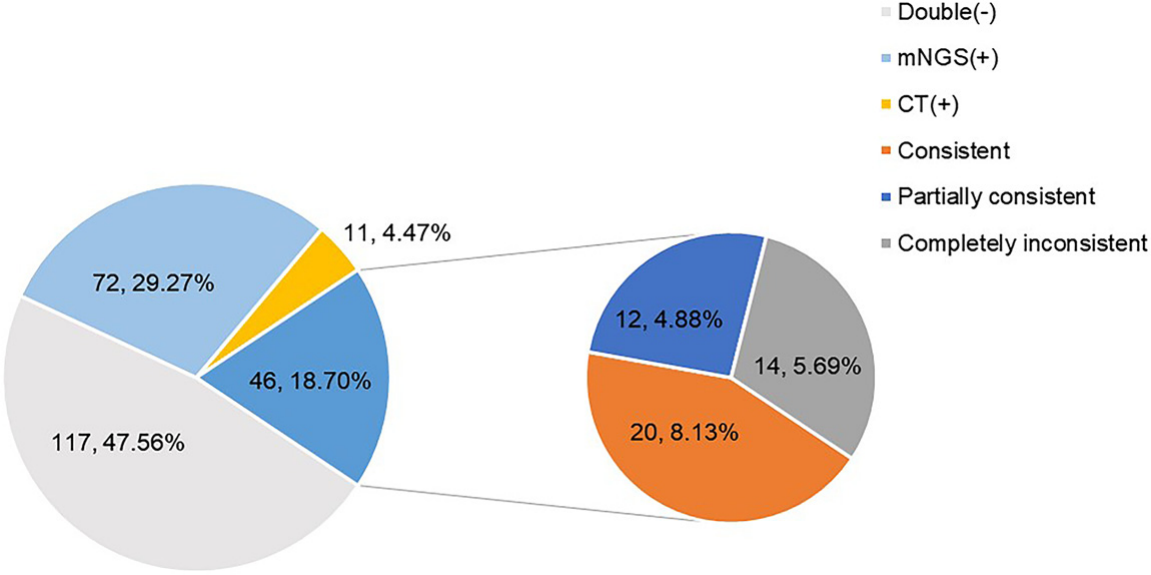
Concordance of diagnosis between mNGS and CT. The results of mNGS and CT were both positive in 46 (46/246, 18.70%) cases. Among the double-positive cases, 20 (20/246, 8.13%) were consistent, 12 (12/246, 4.88%) were partially consistent, and 14 (14/246, 5.69%) were completely inconsistent.

In the study, the results of mNGS and CT were both positive in 46 (46/246, 18.70%) cases and both negative in 117 (117/246, 47.56%) cases. A total of 72 (72/246, 29.27%) cases were positive by mNGS only, but 11 (11/246, 4.47%) cases were positive by CT only. The concordance between the two methods was poor, with a kappa value of 0.31. Additionally, for 46 double-positive cases, the results between mNGS and CT were consistent in 20 (20/246, 8.13%), partially consistent in 12 (12/246, 4.88%), and completely inconsistent in 14 (14/246, 5.69%) (Fig. 2).

### Diagnostic performance of mNGS and CT.

**FIG 3:**
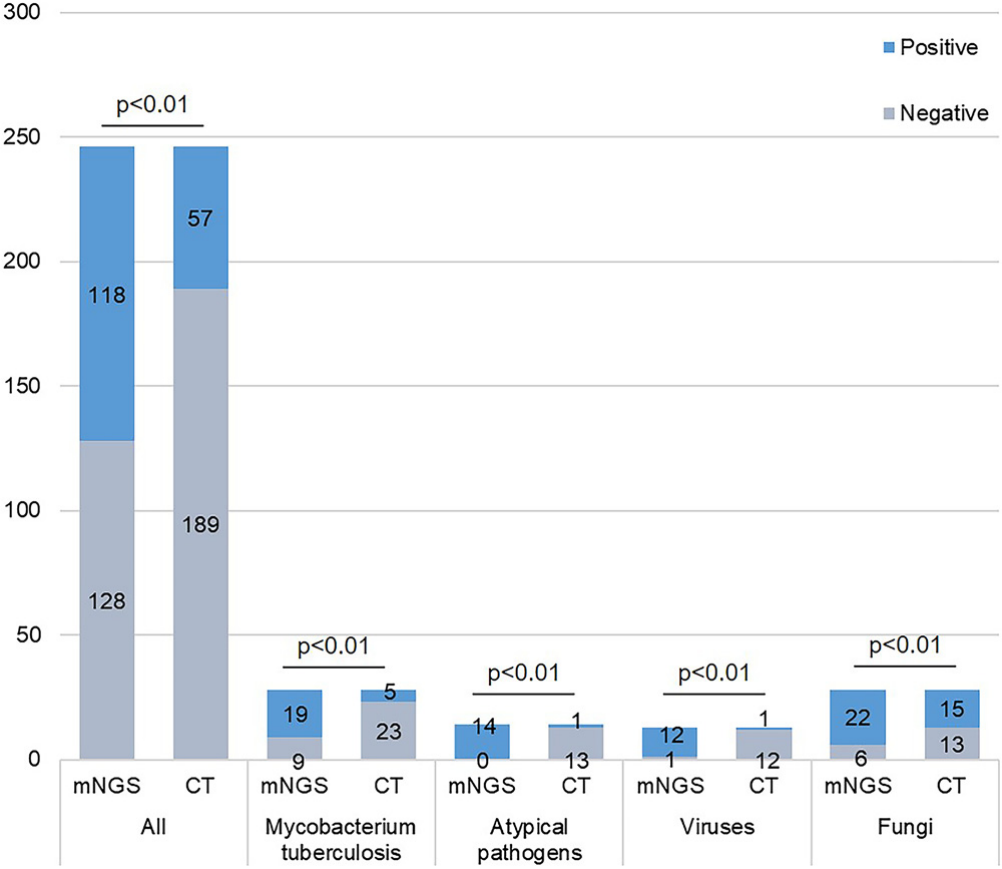
Positive rates of mNGS and CT. Positive numbers of mNGS and CT in suspected pulmonary infection, as well as in the infections of Mycobacterium tuberculosis, atypical pathogens, viruses, and fungi, with *P* < 0.01 being statistically significant.

In this study, the positive rates of mNGS and CT were 47.97% (118/246) and 23.17% (57/246), respectively. Altogether, the positive rate of mNGS was significantly higher than that of CT (*P* < 0.01). In addition, the positive rates of mNGS for M. tuberculosis, atypical pathogen, viral, and fungal infections were 67.86%, 100.00%, 92.31%, and 78.57%, respectively, significantly higher than those of CT (17.86%, 7.14%, 7.69%, 46.43%; *P* < 0.01) (Fig. 3). The sensitivity of mNGS was 53.49% (95% confidence interval [CI]: 46.59% to 60.26%), which was significantly higher than that of CT (23.26%, 95% CI: 17.90% to 29.59%). Additionally, in cases of Mycobacterium tuberculosis, atypical pathogens, viruses, and fungi, the sensitivities of mNGS were also significantly higher than those of CT (67.86% versus 17.86%, *P* < 0.01; 100.00% versus 7.14%, *P* < 0.01; 92.31% versus 7.69%, *P* < 0.01; 78.57% versus 39.29%, *P* < 0.01). The specificities of mNGS and CT were 90.32% (95% CI: 73.10% to 97.47%) and 77.42% (95% CI: 58.46% to 89.72%), respectively, with no statistical difference. In addition, the positive and negative predictive values (PPV and NPV) of mNGS were 97.46% and 21.88%, respectively, with the positive and negative likelihood ratios (PLR and NLR) being 5.53 and 0.51. In comparison, PPV and NPV of CT were 87.72% and 12.70%, respectively, with the PLR and NLR being 1.03 and 0.99 (Table 2).

**TABLE 2:** Diagnostic performance of mNGS and CT in suspected pulmonary infection^*a*^

Assay	Sensitivity, % (95% CI)	Specificity, % (95% CI)	PPV (%)	NPV (%)	PLR	NLR
mNGS	53.49 (46.59–60.26)	90.32 (73.10–97.47)	97.46	21.88	5.53	0.51
CT	23.26 (17.90–29.59)	77.42 (58.46–89.72)	87.72	12.70	1.03	0.99

a mNGS, metagenomic next-generation sequencing; CT, conventional testing; CI, confidence interval; PPV, positive predictive value; NPV, negative predictive value; PLR, positive likelihood ratio; NLR, negative likelihood ratio.

### Pathogens detected by mNGS and CT.

**FIG 4:**
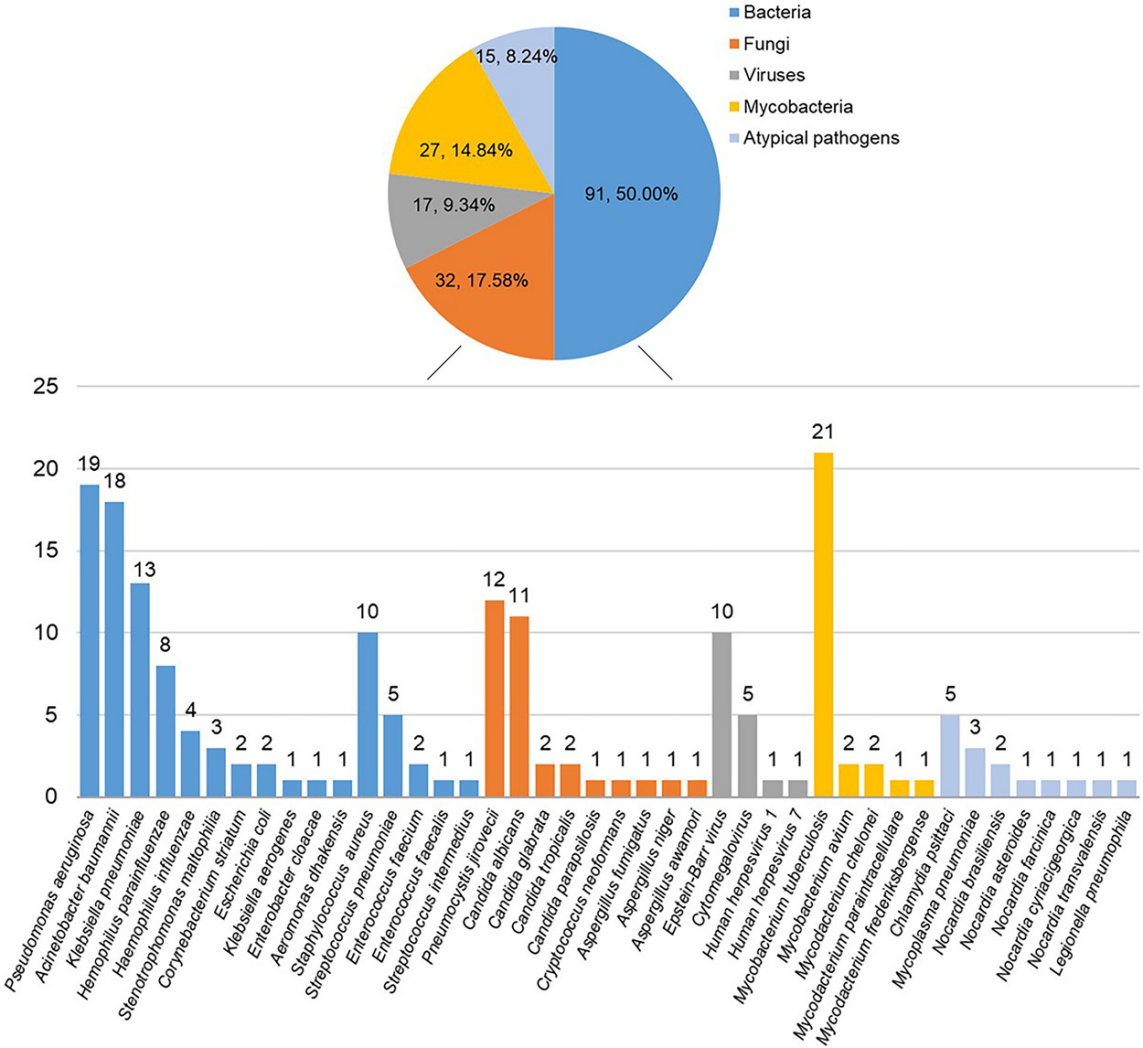
Distribution of pathogens detected by mNGS and CT. Bacteria were the most common pathogens detected by mNGS and CT, followed by fungi, mycobacteria, viruses, and atypical pathogens.

**FIG 5:**
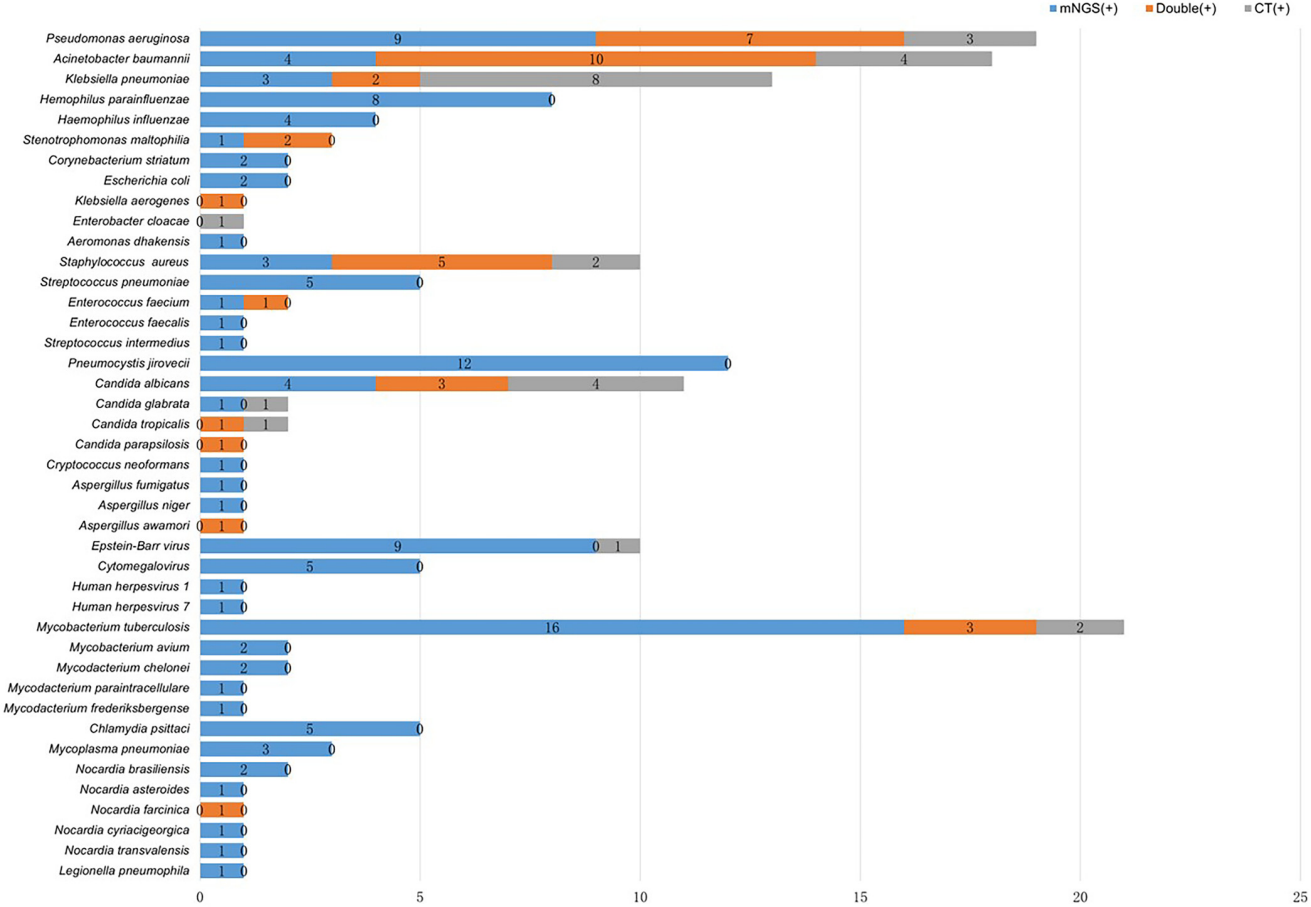
Overlap of pathogens detected by mNGS and CT. Detection efficiency of mNGS and CT for specific pathogens.

A total of 182 strains of pathogens were identified in 125 cases by a combination of mNGS and CT (Fig. 4). Among the microbes isolated, bacteria were the most common pathogens, of which there were 72 (72/182, 39.56%) Gram-negative and 19 (19/182, 10.44%) Gram-positive strains. The most frequently detectable Gram-negative bacteria were Pseudomonas aeruginosa (19/182, 10.44%), followed by Acinetobacter baumannii (18/182, 9.89%) and Klebsiella pneumoniae (13/182, 7.14%). The most common Gram-positive bacteria were Staphylococcus aureus (10/182, 5.49%), followed by Streptococcus pneumoniae (5/182, 2.75%). A total of 32 fungi were identified in 24 cases, of which the most frequent were Pneumocystis jirovecii (12/182, 6.59%) and Candida albicans (11/182, 6.04%). Seventeen viruses were confirmed in 13 cases and the most common was Epstein-Barr virus (10/182, 5.49%), followed by *Cytomegalovirus* (5/182, 2.75%). *Mycobacteria* were detected in 27 cases, with the majority being Mycobacterium tuberculosis (21/182, 11.54%), accompanying a minority of nontuberculous mycobacteria (6/182, 3.30%). A total of 15 atypical pathogens were confirmed in 14 cases, including Chlamydia psittaci (5/182, 2.75%), *Nocardia* (6/182, 3.30%), Mycoplasma pneumoniae (3/182, 1.65%), and Legionella pneumophila (1/182, 0.55%) (Fig. 5 and Data Set S1). Pathogens identified by CT and mNGS of each patient are shown in Data Set S2.

### Characteristics of single infection and mixed infection.

Of all 215 cases of confirmed pulmonary infection, a total of 92 (92/215, 42.79%) were ultimately diagnosed as single infection and 33 (33/215, 15.35%) were diagnosed as mixed infection with at least two pathogens. The remaining 90 (90/215, 41.86%) cases were also comprehensively diagnosed as pulmonary infection with no definite pathogens. Among the mixed infections, the most common combinations were bacteria-bacteria (9/33, 27.27%) and bacteria-fungi (9/33, 27.27%), followed by bacteria-viruses (4/33, 12.12%). In addition, one case was diagnosed as bacteria-fungi-viruses-atypical pathogens coinfection. The median age (*P* = 0.039) and hospital stay (*P* = 0.005) were significantly greater in the cases of mixed infection than in the cases of single infection. There was no significant difference in terms of chronic obstructive pulmonary diseases, bronchiectasis, asthma, diabetes, and tumors between the two groups (Table 3).

**TABLE 3:** Clinical characteristics of single infection and mixed infection^*a*^

Characteristic	Single infection (*n* = 92)	Mixed infection (*n* = 33)	*P*
Age (yr), median (Q1, Q3)	60 (52, 69)	60 (53, 69)	0.039^*b*^
COPD (*n*)	11	2	0.536
Bronchiectasis (*n*)	16	2	0.193
Asthma (*n*)	5	0	0.324
Diabetes (*n*)	15	6	0.805
Tumor (*n*)	14	8	0.243
Hospital stay duration (days), median (Q1, Q3)	14 (10, 19)	14 (10, 20)	0.005^*b*^

a COPD, chronic obstructive pulmonary disease; Q1, first quartile; Q3, third quartile.

b Statistically significant.

### Impacts of mNGS on diagnosis and treatment.

In this study, the final diagnoses of 98 (98/246, 39.84%) cases were modified, but those of 148 (148/246, 60.16%) cases were not. Among the revised 98 cases, 65 were comprehensively diagnosed based on symptoms, signs, medical history, imaging examinations, and the results of CT and mNGS, while only 1 case was diagnosed by CT. The remaining 32 (32/246, 13.01%) cases were diagnosed by mNGS only, most of which were infected with M. tuberculosis (15/32, 46.88%) and atypical pathogens (7/32, 21.88%). Besides this, the turnaround time of mNGS was about 2 days, while that of CT was about 5 days. Additionally, the turnaround time of mNGS was relatively stable, whereas that of CT was not stable and largely depended on the pathogen. In terms of treatment, a total of 138 cases were adjusted, and the remaining 108 cases were not. In most of the cases without adjustment, treatment was not adjusted because of prior empirical medications covering the pathogens detected. The mNGS results directly guided medication in 23 (23/246, 9.35%) cases, which were mostly infected with M. tuberculosis. A total of 113 (113/246, 45.93%) cases were adjusted based on comprehensive condition, and the other 2 (2/246, 0.81%) cases were adjusted by CT only.

## DISCUSSION

In this study, we explored the diagnostic performance of mNGS in suspected pulmonary infection. BALF was taken for mNGS and CT in this study due to its reliability for pathogen detection in suspected pulmonary infection, ensuring the stability of results (17). A total of 246 BALF samples were eventually included in this study, which to date is the first multicenter, large-scale retrospective study based entirely on BALF for pathogen detection by mNGS.

The study showed that the overall positive rate of mNGS was significantly higher than that of CT, especially in infections of M. tuberculosis, atypical pathogens, viruses, and fungi. The sensitivity and specificity of mNGS were also higher than those of CT, especially the sensitivity, which was consistent with the findings of a prior study (18). The sensitivity of mNGS has been over 80% in several studies (19–21), but it was only 53.49% in this study. The primary explanation for this is that all the cases enrolled were of suspected pulmonary infection instead of confirmed pulmonary infection, which was also discussed in a prior study (7). Furthermore, mNGS showed greater advantages in identifying M. tuberculosis, nontuberculous mycobacteria, atypical pathogens, viruses, and fungi. The culture cycle of M. tuberculosis is long, and most species of nontuberculous mycobacteria are difficult to culture (22). Thus, mNGS could be an effective method to identify mycobacteria due to its superior sensitivity (23, 24). C. psittaci, *Nocardia*, *Legionella*, and *Mycoplasma* were classified as atypical pathogens, and were difficult to detect by CT. In recent years, increasing cases of pulmonary infection caused by atypical pathogens have been reported with the clinical application of mNGS (25–28). Our study also confirmed the unique advantages of mNGS in identifying atypical pathogens. Viruses were considered important to the etiology of hospitalized patients with unexplained pulmonary infection (29). PCR for nucleic acid detection improved the diagnosis of virus infections, but it was limited by the large number of types and subtypes of viruses (30). Nevertheless, emerging mNGS with broad detection spectrum and no prediction solves the difficulty of virus detection. Pneumocystis pneumonia is an opportunistic infection with increasing incidence in immunocompromised patients, and its diagnosis by CT is challenging. Fortunately, mNGS greatly improved the diagnosis of Pneumocystis pneumonia (31, 32). Notably, in this study, most of the cases had been exposed to empirical antibiotics before sampling. Prior antibiotic exposure could significantly reduce the sensitivity of CT, but not that of mNGS (7, 33), which partly explained why the sensitivity of mNGS was significantly higher than that of CT in our study. Despite the lack of statistical difference, the specificity of mNGS was higher than that of CT; this was attributed to the high false-positive rate of CT due to contamination by Candida albicans, which is widely distributed in the environment. The quality of the samples seriously affects testing and analysis results, a reminder for clinicians to follow sterile principles during sampling, even though the lower respiratory tract is not sterile (34). In addition, the entire testing process, not just sampling, should be strictly conducted in accordance with standards, improving the reliability of results. In addition, the PPV, NPV, PLR, and NLR of mNGS were also superior to those of CT in our study.

In this study, a total of 92 cases of single infection and 33 cases of mixed infection were finally confirmed. Compared with CT, mNGS detected more pathogens, especially in cases of mixed infection. Additionally, previous studies have reported that the competition between certain microorganisms partly explained the difficulty of CT in detecting multiple pathogens simultaneously (21, 35). Thus, mNGS can comprehensively reveal pathogen distribution in pulmonary infection. Consistent with the results of a previous study (36), the most common type of mixed infection was bacteria-bacteria coinfection. In addition, in this study, the most complicated mixed infection was bacteria-fungi-viruses-atypical pathogens coinfection, in only 1 case, for which CT mainly detected bacteria and fungi while mNGS supplemented the detection of viruses and atypical pathogens. The patient was in critical condition, with severe comorbidities and suppressed immunity, but was improved and discharged with targeted treatment based on the results of mNGS and CT. This reminds clinicians to conduct mNGS testing in a timely manner for critical patients who may have mixed infections with atypical pathogens or viruses, improving their prognosis.

mNGS showed outstanding performance and reliability in the diagnosis of suspected pulmonary infection. Although the final diagnosis and treatment adjustment of cases still mostly depended on the comprehensive results of mNGS, CT, other examinations, and clinical features in our study, modification and adjustment were performed entirely based on the results of mNGS in 32 (32/246, 13.01%) and 23 (23/246, 9.35%) cases, respectively, and had positive impacts on the course and prognosis of these individuals. Additionally, these cases were mostly infected with Mycobacterium tuberculosis, nontuberculous mycobacteria, or atypical pathogens. It is clear that the application of mNGS in clinical practice is challenging. Currently, the implementation of mNGS lacks unified standards, including indications of mNGS, appropriate sampling times, quality control, sequencing platforms, data analysis, and interpretation of results. In addition, it is confoundedly difficult to interpret the results of mNGS; the primary issue is distinguishing between colonization and infection, especially opportunistic infection. Considering individualized clinical conditions, it is impractical to use fixed standards to determine whether a case is a specific microbial infection, especially in cases of mixed infection with multiple pathogens. A previous study distinguished pathogens from colonizing microorganisms via using customized bioinformatics pipelines (37). Subsequently, two models based on mNGS data of respiratory tract samples were developed and validated by cohort. Although the accuracy of the two models was as high as 95.50%, further validation would be necessary due to the small sample size (38). Additionally, mNGS is expensive and is not included in national health insurance; this limits the preferential choice of mNGS, resulting in delayed condition and treatment. Effectively reducing the cost of mNGS or including it in national health insurance will benefit patients.

This study was not without limits. First, the final diagnosis was determined by two or three clinical experts based on the comprehensive condition of patients, but subjective bias was inevitable. Second, the study focused on suspected pulmonary infection, making it difficult to generalize the conclusion to all infectious diseases. Third, as this was a retrospective study, information bias could exist. Thus, we are performing a prospective, multicenter, and large-scale study on the clinical application of mNGS in infectious diseases.

In summary, we systematically analyzed and compared the diagnostic performances of mNGS and CT in suspected pulmonary infection. The results showed that mNGS significantly improved the diagnosis of suspected pulmonary infection, especially M. tuberculosis, atypical pathogen, viral, and fungal infection, and described the pathogen distribution of pulmonary infections in the real world, making it a promising method for microbiological detection and diagnosis.

## MATERIALS AND METHODS

### Study patients.

We performed the retrospective study on 400 BALF samples for mNGS and CT from 4 medical institutions (The Second Affiliated Hospital of Nanjing Medical University; The Second Affiliated Hospital of Nantong University; The First Affiliated Hospital of Nanjing Medical University; The First Affiliated Hospital of Soochow University) in China between January 2019 and September 2021. Researchers at each institution conducted a comprehensive review of clinical data of their patients with suspected pulmonary infection who underwent mNGS and CT. The study was approved by the ethics committee. The data in this study were anonymously obtained and no informed consent was required.

The patient inclusion criteria are as follows: (i) patients with suspected pulmonary infection agreed to undergo bronchoscopy to collect BALF, and both mNGS and CT were performed to detect pathogens; (ii) the quality inspection and BALF sample testing process met the standards of mNGS; (iii) the patient clinical data were complete; and (iv) the raw mNGS sequence data were complete.

The exclusion criteria are as follows: (i) patients refused bronchoscopy and mNGS; (ii) the quality of BALF samples did not meet the standards of mNGS; (iii) the patient clinical data were incomplete; and (iv) the raw mNGS sequence data were incomplete.

### Sample preparation.

An experienced clinician performed bronchoscopy for patients with suspected pulmonary infection to collect BALF for mNGS and CT, according to standard procedures. In brief, the nasal or oral cavities of patients were cleaned with normal saline before bronchoscopy. Subsequently, patients were sedated with dexmedetomidine before the bronchoscopy and anesthetized locally with 2% lidocaine during the examination. All bronchi were examined in detail by electronic bronchoscope, and the lesions shown on a chest computed tomography scan were brush-examined, followed by bronchoalveolar lavage collection. The lavage area was determined according to the lesion area shown on the chest computed tomography scan. If scattered lesions were present, BALF was collected from the right middle lobe or the subsegment of the left lingual lobe (19). A total of about 100 mL (20 mL each time) of sterile normal saline was injected into the target bronchus in batches at 37°C, of which the first 20 mL was discarded to avoid contamination and approximately 5 mL was retrieved into sterile tubes. The BALF samples were divided into aliquots for pathogen detection, and one aliquot was inactivated (56°C, 30 min) before nucleic acid extraction. In addition, sputum and some blood samples from some patients were also collected for CT. Samples of 3 mL sputum from each patient were collected in sterile tubes and were liquefied with 0.1% dithiothreitol for 30 min at room temperature. A total of 58 blood samples were collected in anticoagulant tubes containing ethylenediaminetetraacetic acid and DNA protective agent (Cell-Free DNA Storage Tube, CWY056) and were stored at room temperature.

### Conventional testing.

Conventional testing included bacterial, mycobacterial, and fungal culture and smears: acid-fast staining for *Mycobacteria*, Grocott-methenamine staining for Pneumocystis jirovecii, modified acid-fast bacilli staining for *Nocardia*, and PCR assays and serological antibody detection for Chlamydia pneumoniae, M. pneumoniae, L. pneumophila, Epstein-Barr virus, *Cytomegalovirus*, and other herpes simplex viruses. Additionally, 1,3-β-d-glucan, galactomannan, and Cryptococcus antigen tests were performed for *Candida*, Aspergillus, and Cryptococcus, respectively. T-spot and Xpert testing was done for M. tuberculosis.

### Nucleic acid extraction.

A total of 0.5 mL BALF and 1 g beads with a diameter of 0.5 mm were collected into a 1.5-mL microcentrifuge tube and placed on a horizontal platform on the vortex mixer. Next, the tube was agitated vigorously at 2,800 to 3,200 rpm for 30 min. Subsequently, 0.3 mL BALF was transferred into a new microcentrifuge tube and DNA was extracted using the TIANamp Micro DNA kit (DP316, Tiangen Biotech) according to the manufacturer’s recommendation. RNA was extracted using a QIAamp Viral RNA minikit (52906, Qiagen, China) (13, 39).

**Library construction and sequencing.** DNA libraries were established by DNA fragmentation, end repair, adaptor ligation, and PCR amplification. Briefly, the DNA was sonicated into about 150-bp fragments, which were subjected to terminal repair, phosphorylation, and A-tailing reaction. Subsequently, the sequencing adaptors were added and ligated to the A-tailed fragments. Next, the ligated DNA fragments were purified by magnetic beads and amplified by PCR. RNA was reverse-transcribed and synthesized to DNA using a SuperScript II Reverse Transcription kit (18064-014, Invitrogen, China) (13). Subsequent procedures were the same as those for DNA library construction described above. An Agilent 2100 Bioanalyzer (Agilent Technologies, Santa Clara, California) was used for quality control of libraries. The single-stranded circular DNA libraries were constructed via DNA denaturation and circularization and were subsequently used to generate DNA nanoballs (DNBs) through rolling circle amplification. A BGISEQ-500 platform (Beijing Institute of Genomics, Nanjing, China) was used to sequence qualified DNBs (36, 39).

**Bioinformatic analyses.** First, we removed low-quality reads with short lengths (<35 bp) to obtain high-quality sequence data (20). Second, human sequence data were identified and excluded by mapping on the human reference (hg19) through Burrows Wheeler Aligner software (40). Finally, the remaining data were compared with the microbial genome database (http://ftp.ncbi.nlm.nih.gov/genomes/) by Burrows Wheeler Aligner software (0.7.17-r1188). The database was downloaded from the National Center for Biotechnology Information and contained 11,910 bacteria, 7,103 viruses, 1,046 fungi, and 305 parasites related to human diseases (35).

**Criteria for positive mNGS results.** The mNGS positive standards in this study were established according to those in the literature due to the differences in sequencing platforms and the lack of unified standards for interpreting mNGS results (15, 19).

1. The relative abundance of pathogens detected by mNGS at the genus level was greater than or equal to 30%, regardless of the CT results.

2. mNGS and CT detected the same microorganism and the unique sequence from a single species was greater than or equal to 50.

3. M. tuberculosis was considered positive when at least 1 read was mapped to either the species or the genus level.

Notably, it was insufficient to judge whether microorganisms were infected, colonized, or contaminated according to the results of mNGS and CT only. Two experienced clinical experts jointly determined pathogens according to clinical characteristics and the results of mNGS and CT. In cases of dissent, the third expert would adjudicate.

### Statistical analysis.

Different types of data were described by corresponding representation methods, such as median and percentage, for statistical analysis. The SPSS 23.0 software (IBM, Armonk, NY, USA.) was used for data analysis, and a two-tailed *P* value of 0.05 was considered statistically significant.

### Data availability.

The sequence data are available in the NCBI SRA under NCBI BioProject ID PRJNA845064.
